# An e-leadership training academy for practicing clinicians in primary care and public health settings

**DOI:** 10.1017/cts.2020.574

**Published:** 2021-01-05

**Authors:** Erin E. Sullivan, Dena Moftah, PaMalick Mbye, Taylor Weilnau, Jonathan N. Tobin

**Affiliations:** 1 Center for Primary Care, Harvard Medical School, Boston, MA, USA; 2 Center of Excellence for Primary Care Practice-Based Research and Learning, Clinical Directors Network, Inc. (CDN), New York, NY, USA; 3 Center for Clinical and Translational Science, The Rockefeller University, New York, NY, USA

**Keywords:** Primary care, leadership, interprofessional education, community health

## Abstract

Leadership is an essential competency for clinicians; however, these skills are not a standard part of health professionals’ education and training. Access to resources (time, money) is frequently cited as a barrier for clinicians to participate in leadership development programs. We aimed to tackle this barrier within postgraduate health professions education and training through establishing an online e-Leadership Academy. The e-Leadership Academy was developed as a community–academic partnership between Clinical Directors Network, Inc. (CDN) and the Harvard Medical School Center for Primary Care to train clinicians and healthcare staff in the fundamental concepts and skills for leading within a clinical practice. For this article, the primary outcome analysis examined participants’ responses to both formative and summative evaluations that took place throughout and at the end of the course. Results were used to assess course quality, participant satisfaction, participant engagement, and provide recommendations about future course offerings for a similar audience. The authors propose that future training programs could measure the changes in team behavior and clinical outcomes using expanded evaluations. Proposed plans for expansion of the e-Leadership Academy include developing additional modules, virtual coaching and mentoring, and the potential integration of an in-person component.

## Problem

Leadership is widely recognized as an essential competency for clinicians in order to provide accessible, high-quality health care, and promote collaborative change within the healthcare system. Health care has lagged behind many other industries in terms of leadership training despite this consensus [1,2]. Since 2012, peer-reviewed literature shows a marked increase in papers discussing clinician leaders. Studies have also demonstrated that training clinician leaders improves professional satisfaction [3]. Courses to train clinician leaders have expanded in response to increasing demand. This is an encouraging development given that health care tends to select leaders based on demonstrated clinical excellence or scientific innovation, but these forms of success do not necessarily translate into strong leadership proficiency for clinicians [4].

The barriers to adding leadership development to already packed professional school curricula are numerous and well documented [5,6]. For example, at medical schools, leadership training tends to fall into three categories: (1) dual degree programs (MD/MBA); (2) longitudinal tracks with a leadership focus; and (3) classes that range from a one-off session often consisting of autobiographical soliloquies to multiple, months-long components using case-based learning. The Association of American Medical Colleges (AAMC) published explicit entrustable professional activities, or competencies for medical school graduates regarding the ability to collaborate on an interprofessional team [7]. This led to a medical education curriculum redesign, which emphasizes team-based care, including teamwork, team leadership, and change in leadership skills [8].

There are a growing number of continuing education leadership training opportunities for clinicians at varied career stages. In most of these examples, trainees opt in to the leadership development track or course, which increases the likelihood that participants are actively seeking a management or leadership-oriented career. It does not account for those who might become “accidental” or “volunTOLD” leaders at some point in their career. For example, after a medical director left the practice, a participant found himself taking on the role, as there were no other available or willing physicians to do it. Those who find themselves in new, and sometimes unexpected roles, often look for just-in-time leadership training to support them [9].

As noted, there are significant barriers and issues with leadership training across all levels of training. A major barrier is an access to resources for training, where resources are defined as money and time [9,10]. In this paper, we aim to describe a novel community-academic online clinical leadership training program, which aims to make leadership training content more relevant and accessible.

## Approach

The Harvard/Clinical Directors Network (CDN) e-Leadership Academy was developed as a community–academic partnership between the Harvard Medical School Center for Primary Care and CDN in 2018, after a successful three-webinar pilot series in 2017–2018.

CDN is a not-for-profit clinician membership organization, practice-based research network (PBRN), and clinician training organization. CDN provides peer-based activities for clinicians practicing in Federally Qualified Health Centers (FQHCs) and other safety net primary care practices serving low-income, minority, and other under-resourced communities. CDN’s goal is to translate clinical research into clinical practice for the enhancement of health equity and improvement of public health. Founded as a clinical leadership training organization and PBRN based on the principle of peer-to-peer learning and support, CDN originally presented in-person clinical leadership training through conferences and workshops in the 1990s [9], and then migrated to online CME-accredited webcasts in 2000. CDN has designed and conducted over 1000 online CME-accredited programs in collaboration with other institutions designed to engage and encourage clinical leaders and team members to critically examine their delivery of care. Most of these programs carry enduring CME credits and are available on demand from the CDN Webcast Library (https://www.CDNetwork.org/Library).

The Harvard Medical School Center for Primary Care (https://primarycare.hms.harvard.edu/) works to strengthen health care through the transformation of systems, teams, and leaders. Developing leaders within primary care has been a core part of the Center’s mission since its inception in 2011. The Center’s leadership development portfolio includes programs that train leaders at various career stages, from medical students through health system executives [11,12]. The partnership with CDN presented an opportunity for the Center to repurpose and digitize its basic leadership development content for an online and national audience of safety net, health center, and health department clinicians and staff.

This community–academic partnership aligned with many of CDN’s founding principles, which include supporting clinical networks to create professionally satisfying work experiences for clinicians working in underserved areas. CDN has also provided managerial training in response to needs articulated by clinicians in their network since 1986 [9]. Furthermore, professional satisfaction and improved clinician retention have also been linked to leadership training [3,13].

As this was a community–academic partnership, CDN and Harvard’s Center for Primary Care conceptualized the online program with input from community-based clinicians and other stakeholders, actively soliciting feedback about content while the program was under development and being delivered. CDN and the Center for Primary Care ensured a sustainability and dissemination plan to make the content accessible beyond the end of the “live” version of the program in an asynchronous on-demand format [14].

The result of the collaboration was a 100% virtual leadership academy, offered over a 10-month period that covered the fundamental concepts and skills for leading within a clinical practice. We aimed to reach a broader audience with limited time, ability to travel, and financial resources to pay for training. The audience for this program was mainly FQHCs, other community health centers, PBRNs, health departments, and clinical and translational science award grantees across the USA. It is unlikely that these typically resource-constrained organizations would be able to afford sending an entire team to an in-person leadership development program, but the lower cost of distance learning makes it more feasible to support teams in attending a monthly webinar.

Given the interprofessional nature of healthcare teams, we aimed to be as inclusive as possible in participant recruitment. The course was promoted as appropriate for current or aspiring clinical leaders from a variety of backgrounds: physicians, nurse practitioners, physician’s assistants, nurses, pharmacists, administrators, and social workers. Training is often ineffective if only one individual is trained [15], and data supporting team training’s efficacy have been increasing over the past 7 years [16–18]. While the evidence base on the efficacy of team training in non-acute settings lags behind the evidence base for acute settings, the overall evidence on team training’s impact is growing; in particular, the evidence demonstrates that team training can improve team processes, patient safety, and organizational outcomes. Further, team training is effective at any career stage [18], thus, we encouraged entire teams to register and participate together in the e-Leadership Academy and a multi-registrant discounted pricing structure was designed to encourage team participation. As the course was accredited for 15 CME credits, many participants used funds from their CME budgets to support team participation.

Studies on clinician leadership development currently lack a consensus on the core skills and competencies clinicians need to lead, such as problem-solving skills and communication skills, high degrees of emotional intelligence, and a deep understanding of topics like quality improvement, payment, and health systems [19]. We opted to design the curriculum for the e-Leadership Academy based on the Harvard Center for Primary Care’s in-person Leadership Academies that were held in the greater Boston area. In building the e-Leadership Academy, we repurposed existing content from the Center’s in-person physician leadership trainings. We presented the existing in-person curriculum to a representative group of clinicians from FQHCs who are part of CDN’s Board of Directors, in order to receive feedback on course content during the development phase. We also provided an initial course session in which we were able to provide an overview of the program and get feedback from potential participants.

The overarching goal was for participants to learn the fundamental concepts and skills for leading oneself, a team, and change within clinical practice. We created a 10-month series comprised of a monthly 90-minute webinar (see Table 1). The 10 sessions were divided into 3 specific modules, each with their own key objectives:

1. Leadership Concepts (sessions 1–3): Participants will understand fundamental concepts related to leadership and identify their own specific leadership style.
2. Leading Teams (sessions 4–7): Participants will learn how to build and maintain an effective team; develop strategies for hiring and performance evaluation; and optimize communication and have difficult conversations.
3. Leading Change (sessions 8–10): Participants will evaluate frameworks for leading change; assess challenges in responding to change due to the external environment or internal initiatives; and build strategies for resilience and joy in work in a constantly changing healthcare landscape.

**Table 1.** Harvard/Clinical Directors Network, Inc. (CDN) clinical leadership virtual academy course curriculum

Session title	Topics	Learning Objectives *(After this session, participants will be able to…)*
Module 1: Leadership concepts		
What do leaders do?	Leadership versus management Core leadership competencies Setting your leadership vision	Describe the difference between a leader and a manager Articulate a leader’s role within a healthcare organization Define what a vision is and how successful leaders determine and communicate their vision
Understanding different leadership styles	Goleman’s six styles of leadership Impact of leadership style VIA character strengths survey for self-assessment	Identify examples of each of Goleman’s six leadership styles Understand the advantages and disadvantages of each of Goleman’s six leadership styles Analyze situations in which one leadership style might be more effective than others
The “Es” of Leadership	Emotional intelligence Emotional agility Emotional courage	Describe emotional intelligence, emotional agility, and emotional courage Articulate why these concepts are important for effective and strong leadership Learn and apply strategies for improving these concepts as a leader
Module 2: Leading teams		
Building a team	Team definition Team effectiveness Team dysfunction	Describe a team and distinguish true teams from groups of individuals Understand building, participating in, and leading effective teams Identify the common dysfunctions of a team
Hiring strategies	Hiring process Best practices for onboarding Recruiting a diverse workforce	Develop a complete hiring process from job descriptions through to extending an offer Articulate best practices for the onboarding process Identify tools and techniques for recruiting and hiring a more diverse workforce
Optimizing the team	Day-to-day team management Best practices for meetings Giving and receiving feedback Team problem-solving	Construct better meetings and 1:1s for advancing teamwork, projects, and efficiency Articulate strategies for giving and receiving feedback Apply team problem-solving techniques
Managing conflict and difficult conversations	Elements of a difficult conversation Framework for difficult conversations Managing and resolving team conflict	Apply strategies for productively managing conflict within your team Identify communication strategies for difficult conversations Increase confidence in handling conflict and difficult conversations
Module 3: Leading change		
Creating a culture of improvement	Quality improvement Fixed versus growth mindset	Describe common quality improvement tools and techniques Articulate the difference between a fixed and growth mindset Identify concrete strategies to build quality improvement principles into your daily work.
Leading a changing and diverse workforce	Diversity challenges for teams and organizations Unconscious bias Strategies for leading a diverse workforce	Understand the changing nature of the workforce and the challenges associated with these changes Articulate different types of bias that may impact you as a manager or as a leader Identify strategies and tools to make you a steward of diversity in the workforce
Leading a joyful practice	Drivers of burnout Clinician/staff engagement strategies Joy, purpose, and meaning in work	Review the current evidence related to burnout in health care Describe the drivers of burnout and engagement of clinicians and staff Identify leadership strategies to support your team in finding joy in practice

Faculty affiliated with the Harvard Center for Primary Care were recruited based on their willingness to migrate their in-person leadership-related course content to an online format. Faculty were supported in preparing their online materials and planning interactive session activities by the course director and project coordinator. Harvard faculty were responsible for session content, while CDN maintained responsibility for all technical aspects of the course, including the online training infrastructure, CME accreditation, and marketing.

Each session was structured with 50–60 min of content delivery by faculty presenters, drawing heavily on case studies, and included audience engagement activities via polls and chat boxes; chat box responses were summarized in real time and shared with participants using frequency-based word clouds. A 30-minute question and answer session followed didactic content delivery. Participants entered questions into a chat box during the content presentation, and faculty answered questions presented by the CDN moderator. Webinars were held at 12 pm ET on the same day of each month (i.e., second Wednesday) to facilitate participant attendance and faculty participation. Webinars were also available on-demand to all participants so those unable to attend the live sessions were able to attend at their convenience, and participants could review parts or entire sessions again. The complete online course was accredited for up to 15 CME/CNE credits (1.5 credits awarded per session), and carries enduring CME/CNE accreditation so new participants can register and view the full course asynchronously on-demand (www.CDNetwork.org/HarvardLeadership).

## Outcomes

Assessment of the e-Leadership Academy included both formative and summative evaluations. In order to receive CME credit for each session, participants were required to complete a five-question quiz after attending, or viewing on-demand, each monthly session. Additionally, for each session, we included three–four questions related to participant satisfaction, feedback on course content, and feedback on the faculty presenter. For this article, we primarily analyzed the participant responses to the final course survey. The survey was conducted at the conclusion of the course to assess course quality, participant satisfaction, and provide data about future offerings that would be useful to our target audience.

### Participation and Engagement

**Fig. 1.**
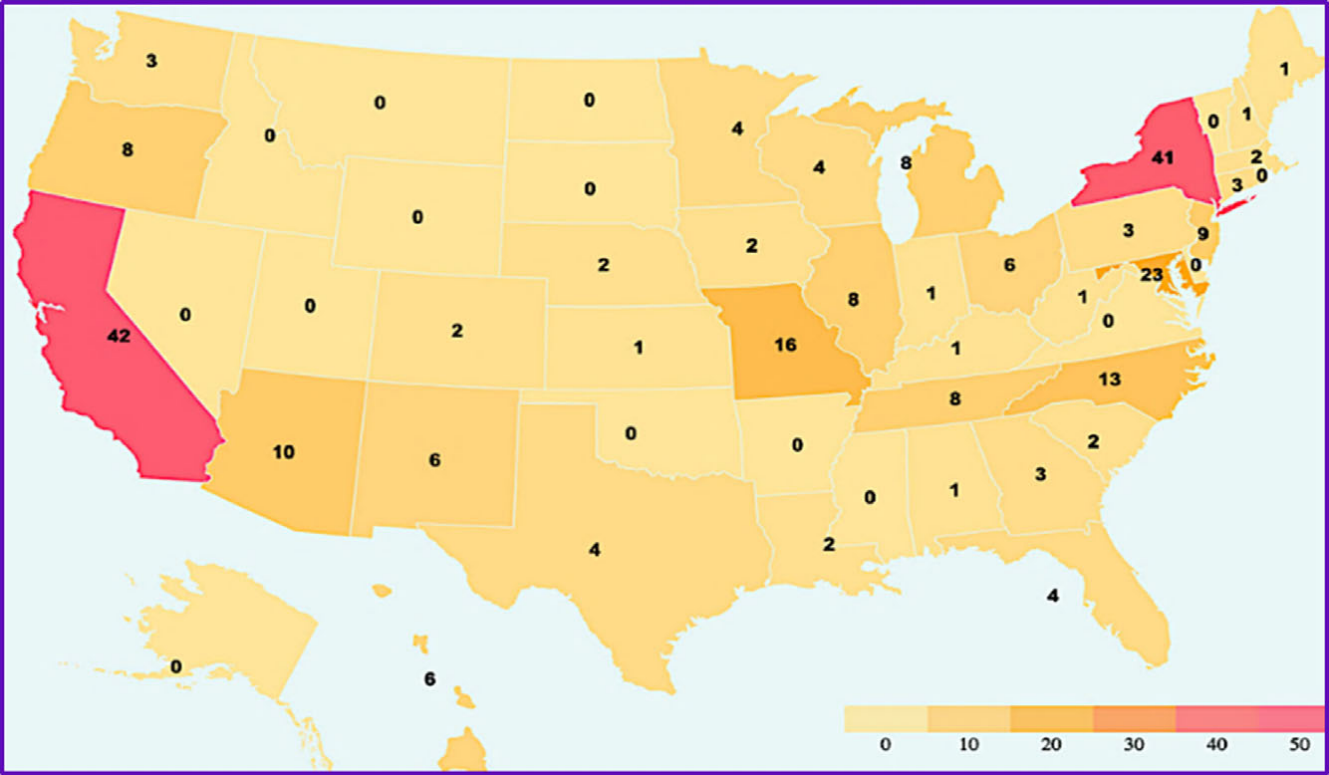
Geographic distribution of registrants.

Two hundred seventy-six people from 36 states/territories participated, the top 3 states being California (16.7%), New York (16.3%), and Maryland (9.1%) (see Fig. 1). The majority (72%) of the cohort registered as part of teams, with 198 teams represented in the program; teams ranged in size from 2 to 9 participants (45%), 10 to 20 participants (19%), or 21+ participants (8%). A total of 78 registrants (28%) enrolled individually, rather than as part of a team. Anecdotally, we know some groups chose to watch and discuss each webinar together as a team, while other groups had their members watch the webinars individually and came together subsequently as a team, either monthly or quarterly, to discuss how to apply the content to their practice. A total of 15 different occupations were represented, with physicians (46%) and administrators (18%) being the largest categories. A total of 97 unique organizations were represented in the program; those with 10 or more registrants included medical schools, health systems, and hospitals.

In addition to survey responses, a more objective method of measuring participant engagement was through the use of Zoom “attention scores.” Attention scores were captured passively and defined as the percent of time a participant had the webinar window active and in the foreground on their device. Conversely, participants were considered “inactive” if they clicked away from the live webinar window to use a different program. Participants displayed a cumulative average attention score of 88% during the course sessions, with a total of 85,364 active minutes of attention to the course programming (see Table 2). On average, 107 (71.3%) participants viewed the webinars live each month, and 43 (28.7%) viewed the on-demand recordings (yielding an average of 150 viewers per month overall).

**Table 2.** Participant attendance and engagement

Session name	Cumulative minutes viewed	Average attention score^*^
Session 1: What do leaders do?	8616	93%
Session 2: Understanding different leadership styles	11,026	98%
Session 3: “The Es” of leadership	9598	88%
Session 4: Building a team	8385	92%
Session 5: Hiring strategies	9903	99%
Session 6: Optimizing the team	7980	86%
Session 7: Managing conflict and difficult conversations	8224	90%
Session 8: Quality improvement: Tools and techniques	4914	65%
Session 9: Leading a changing and diverse workforce	9278	90%
Session 10: Leading a joyful practice	7436	83%
**TOTAL**	**85,364**	**88%**

* Attention scores are calculated by Zoom and it is defined as the percent of time the Zoom webinar window was the active screen on the participant’s device.

### Satisfaction Data

Quantitative data from participant evaluations were collected on a 5-point Likert scale ranging from 1 = Poor to 5 = Excellent and analyzed both during, and at the conclusion of the course. In the final evaluation, 96% of participants reported that they were either “Very Satisfied” or “Satisfied” with the course; 92% said that they were likely to recommend it to a colleague/friend, and 88% indicated that the course content will help their daily practice. Qualitative data provided in response to open-ended questions in the course evaluations demonstrated that participants found the entire course valuable, many reporting sentiments such as “As a new medical director of a primary care clinic, the entire course was valuable,” or “Participating as a team has been exceptionally beneficial to the organization.” Participants also suggested that they would use course content to improve their current work environment, “Hearing a variety of examples and suggestions for ways to improve the work environment was helpful,” or “This course helped me to learn how to listen and to implement changes in a way that will be comfortable to my staff.”

The following aspects of the course were rated as the most valuable: leadership concepts and theories, content related to the work environment (team building, burnout, culture, workforce), and managing difficult situations. Respondents rated the subject-matter organization and delivery on a 5-point Likert scale ranging from 1 = Poor to 5 = Excellent and 99% rated the subject-matter organization and delivery of the course as “Excellent” or “Good”; and 96% of respondents rated the quality of the course as “Excellent” or “Good.”

**Fig. 2.**
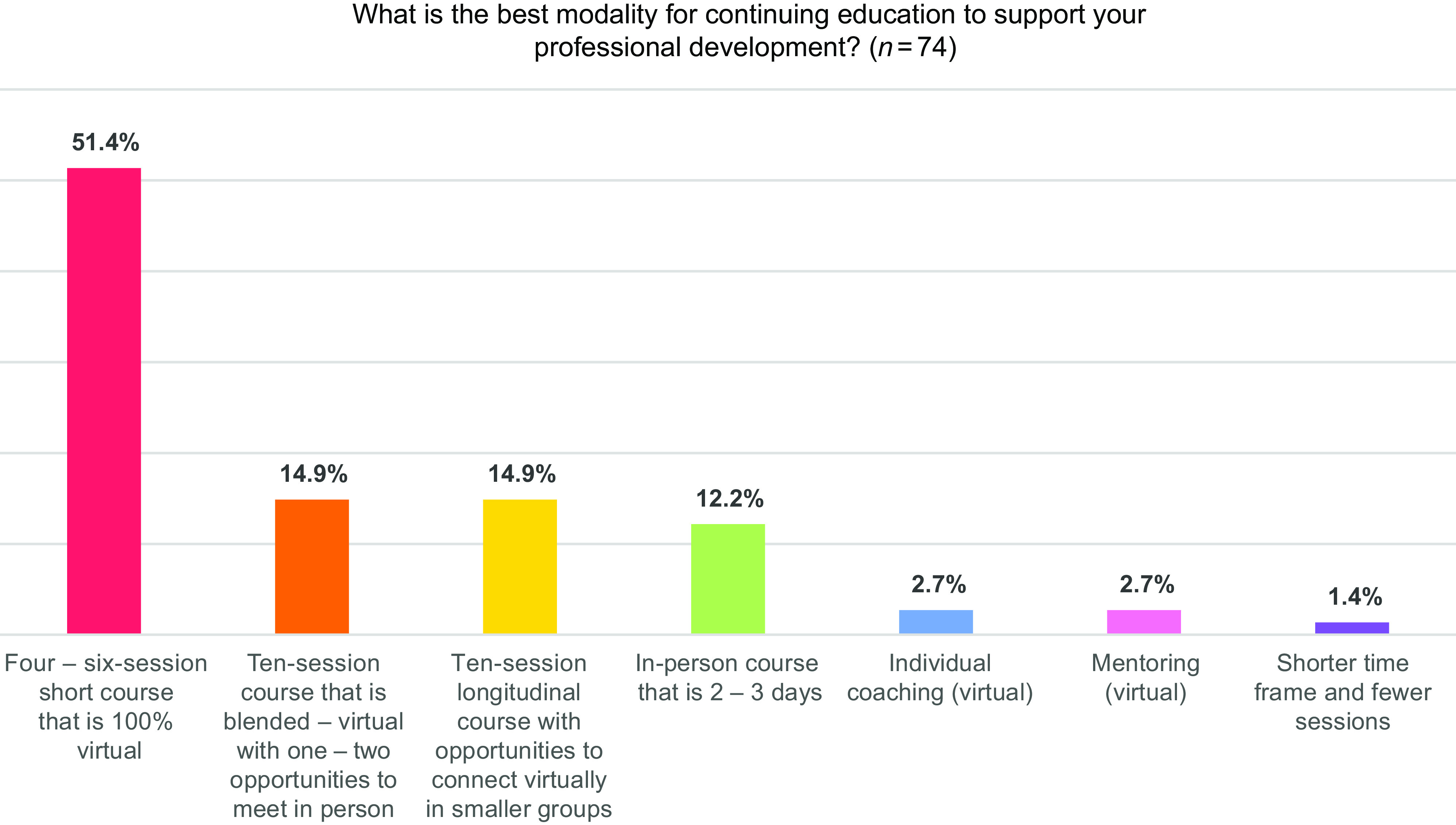
Preferred modality for continuing education.

In the final survey, we asked participants about modalities that best support their professional development goals. Over 50% of respondents reported that they preferred an entirely virtual course (see Fig. 2). We also learned that during the live virtual format, our audience felt connected to each other via the “chat” feature and the Q&A segment of each session, where they reported being able to share insights or learn from other organizations. Participants also found the course accessible, commenting that “once a month is easier to keep up with for someone with a busy schedule” and “having access to the recordings afterward was a great feature for the meetings.”

## Limitations

One limitation is related to our monthly participation rates, which averaged 150 viewers per month (out of 276, 54%). These results may underestimate the true attendance numbers for two specific reasons. First, we know, anecdotally, that members of a team watched the program together at a single computer or via a conference room screen. In those instances, that would only count as one participant view. Second, while all viewers were asked to complete the session evaluation each month, that evaluation was connected to CME credit. If a participant did not need the CME credit, it is possible they did not fill out the evaluation. Finally, another consideration for participation rates relates to the possibility that groups of participants were enrolled by a senior leader, but some of those participants had varied interests or availability to attend the program.

In this preliminary collaboration, we did not collect any independent measures of implementation or impact of the new content in clinical practice. Future training programs could measure the changes in the behavior of teams (process) and evaluate the clinical impact (outcomes) using electronic health records (EHR) data. Nevertheless, the course was designed to provide learners with an opportunity to assimilate what they are learning, try new ways of leading and managing, and reflect on those actions or experiments.

## Next Steps

We plan to sustain and disseminate our current e-Leadership Academy through an online portal, which contains the course content, supplemental reading materials, and tools and templates for participants. While we were able to leverage the existing CME budgets of many FQHCs to offset registration fees, we continue to seek new sources of funding (i.e., novel trainings, philanthropic support) to support additional participant access and expansion of our offerings.

The Harvard/CDN e-Leadership Academy focuses on delivering relevant content so that our clinical audience can acquire new knowledge and team leadership skills. We acknowledge that acquiring new knowledge and skills does not necessarily translate into measurable action. To that end, learners in Year 2 of the e-Leadership Academy will view recorded webinars from Year 1, and complete an activity that allows them to directly apply the learning principles into their practice settings during the webinar. Then, the cohort will attend bimonthly office hours, which are structured to increase participant interaction and engagement with faculty and other learners, and create a space to reflect and seek feedback from faculty and peers. This increases the potential audience and maximizes participant–faculty interaction using “flipped classroom” principles [20].

Future plans for expansion of the e-Leadership Academy include developing additional modules, or short courses, which build on the initial curriculum and provide more advanced, in-depth content tailored to training gaps identified by our audience. Prior to the COVID-19 pandemic, Harvard/CDN was working to identify a way to position the current program as a blended, longitudinal program with an in-person component, such as at the beginning and/or at the end of the series, perhaps conducted during a national conference with high levels of attendance by participants. An in-person component would allow the curriculum to include more interactive activities, such as role plays and simulations, in which participants can practice leading and receive real-time feedback from peers. Given the pandemic and concurrent rapid increase of distance learning options, immediate future plans for expanding the e-Leadership Academy include more individualized options for participants, such as virtual coaching and mentoring to provide real-time support to frontline clinical leaders.

We presented here a reproducible, scalable model for developing, disseminating, and evaluating a community-academic learning collaborative that is enduring and far-reaching, and was well received by a wide range of clinicians and other healthcare workers. The key components of creating the e-Leadership Academy included an emphasis on team-based learning, partnerships, content curation, faculty support and recruitment, marketing and participant recruitment, easy-to-use web-based technology, and ongoing evaluation. We identified several innovations, extensive use of chat to provide a communications platform for participants, and real-time qualitative analysis and feedback to participants using word clouds, in addition to using attention scores. We believe the model is viable, timely, and critical within the context of public health emergencies, such as COVID-19, in providing on-demand content as well as relevant additional short courses that provide just-in-time information for managing and leading during a crisis. The virtual model also allows participants to strengthen their connections to each other and leverage CDN’s long-standing peer support network that remains accessible virtually and beyond the conclusion of any one specific program.

## Supplementary material

For supplementary material accompanying this paper visit http://dx.doi.org/10.1017/cts.2020.574. click here to view supplementary material
