# Supplementary material for: An e-leadership training academy for practicing clinicians in primary care and public health settings
Source: J Clin Transl Sci. 2021 Jan 5;5(1):e83. doi: 10.1017/cts.2020.574 (PMC8111608; doi:10.1017/cts.2020.574)
Supplement: Supplementary file 1 [file S2059866120005749sup001.docx]

**Appendix 1. Sample Participant Course Evaluation Questions**

| **Question** | **Response options** |
| --- | --- |
| How would you rate your overall satisfaction with this course? | Very Satisfied, Satisfied, Neutral, Slightly Satisfied, Not Satisfied |
| How likely are you to recommend this course to a colleague or a friend? | Likely, Not Sure, Not Likely |
| Please list up to three things you found valuable in this course. | Open-ended |
| Please list up to three suggestions of what you would change about this course. | Open-ended |
| Please select your top three favorite sessions and explain why in the comment box. | Select session name, open-ended comments |
| How would you rate the interactive exercises included in the live sessions? | Excellent, Good, Average, Fair, Poor |
| Which instructors were most engaging? | Select faculty name, open-ended comments. |
| How would you rate the subject matter organization and delivery? | Excellent, Good, Average, Fair, Poor |
| How would you rate the user friendliness of the course software? | Excellent, Good, Average, Fair, Poor |
| How would you rate the quality of the course content? | Excellent, Good, Average, Fair, Poor |
| How would you rate the extent to which this course will help you in your daily practice? | Excellent, Good, Average, Fair, Poor |
| What other information or topic area would you like to see in a webcast? | Open-ended |
| What is the best modality for continuing education to support your professional development? | Open-ended |
